# Targeting the Cdc2‐like kinase 2 for overcoming platinum resistance in ovarian cancer

**DOI:** 10.1002/mco2.537

**Published:** 2024-04-13

**Authors:** Yinan Jiang, Shuting Huang, Lan Zhang, Yun Zhou, Wei Zhang, Ting Wan, Haifeng Gu, Yi Ouyang, Xiaojing Zheng, Pingping Liu, Baoyue Pan, Huiling Xiang, Mingxiu Ju, Rongzhen Luo, Weihua Jia, Shenjiao Huang, Jundong Li, Min Zheng

**Affiliations:** ^1^ Department of Gynecology, Sun Yat‐sen University Cancer Center, State Key Laboratory of Oncology in South China Collaborative Innovation Center for Cancer Medicine Guangzhou China; ^2^ Department of Gynecology, Guangdong Provincial People's Hospital Guangdong Academy of Medical Sciences Guangzhou China; ^3^ Department of Radiation Oncology, The Third Affiliated Hospital of Kunming Medical University, Yunnan Cancer Hospital Yunnan Cancer Center Kunming China; ^4^ Department of Clinical Immunology, The Third Affiliated Hospital Sun Yat‐sen University Guangzhou China; ^5^ Department of Radiation Oncology, Sun Yat‐Sen University Cancer Center, State Key Laboratory of Oncology in South China Collaborative Innovation Center for Cancer Medicine Guangzhou China; ^6^ Department of Pathology, Sun Yat‐Sen University Cancer Center, State Key Laboratory of Oncology in South China Collaborative Innovation Center for Cancer Medicine Guangzhou China; ^7^ Department of Experimental Research, Sun Yat‐sen University Cancer Center, State Key Laboratory of Oncology in South China Collaborative Innovation Center for Cancer Medicine Guangzhou China; ^8^ Department of Obstetrics and Gynecology, Guangzhou Women and Children's Medical Center Guangzhou Medical University Guangzhou China

**Keywords:** Cdc2‐like kinase 2, DNA damage repair, ovarian cancer, platinum resistance, synergistic lethal

## Abstract

Platinum resistance represents a major barrier to the survival of patients with ovarian cancer (OC). Cdc2‐like kinase 2 (CLK2) is a major protein kinase associated with oncogenic phenotype and development in some solid tumors. However, the exact role and underlying mechanism of CLK2 in the progression of OC is currently unknown. Using microarray gene expression profiling and immunostaining on OC tissues, we found that CLK2 was upregulated in OC tissues and was associated with a short platinum‐free interval in patients. Functional assays showed that CLK2 protected OC cells from platinum‐induced apoptosis and allowed tumor xenografts to be more resistant to platinum. Mechanistically, CLK2 phosphorylated breast cancer gene 1 (BRCA1) at serine 1423 (Ser1423) to enhance DNA damage repair, resulting in platinum resistance in OC cells. Meanwhile, in OC cells treated with platinum, p38 stabilized CLK2 protein through phosphorylating at threonine 343 of CLK2. Consequently, the combination of CLK2 and poly ADP‐ribose polymerase inhibitors achieved synergistic lethal effect to overcome platinum resistance in patient‐derived xenografts, especially those with wild‐type BRCA1. These findings provide evidence for a potential strategy to overcome platinum resistance in OC patients by targeting CLK2.

## Graphical abstract

Cisplatin‐stimulated p38 phosphorylates Thr343 of CLK2 to increase CLK2 protein stability by blocking its ubiquitination and degradation. Increased CLK2 activates BRCA1 to enhance DNA damage repair and induced resistance to cisplatin. Thus, targeting the CLK2/BRCA1 axis overcomes platinum resistance in ovarian cancer.

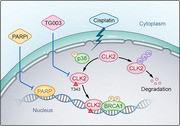

## 1 INTRODUCTION

Ovarian cancer (OC) is one of the most frequently diagnosed cancers in women and the leading cause of gynecologic cancer‐related death worldwide, with 314,000 incidence cases and 207,000 deaths in 2020.^1^, ^2^ Due to the asymptomatic initial stage and a lack of effective screening methods, the disease is often advanced at the time of diagnosis. Currently, surgical debulking with platinum‐based chemotherapy is the cornerstone treatment for advanced OC.^1^, ^3^, ^4^ Although the majority of patients achieve complete response after primary treatment, approximately 65−80% will recur within 3 years,^5^, ^6^ and the 10‐year survival was only 17%.^7^ Platinum‐free interval (PFI), defined as the duration of response to prior platinum‐based chemotherapy, is the most widely used predictor of response to subsequent treatment.^8^ Patients are considered platinum resistant (PR) when their PFI is less than 6 months and may exhibit low response rates to the secondary treatment. Given the significant relation between platinum resistance and poor survival, there is an urgent need to elucidate the mechanisms of platinum resistance and discover new targets for the treatment of OC patients.

Scientific studies have demonstrated that homologous recombination (HR) deficiency results in elevated genome instability, which increases OC cell's response to platinum‐based chemotherapy and develops anticancer strategies such as poly ADP‐ribose polymerase (PARP) inhibitors.^9^ Breast cancer gene 1 and/or gene 2 (BRCA1 and/or BRCA2) mutations are the strongest known mechanisms of HR deficiency and PARP inhibitors have become the standard maintenance treatment for advanced OC patients with BRCA mutations.^10^ However, approximately 85% OC patients with BRCA wild‐type (WT) are not currently candidates for PARP inhibitors. An encouraging approach is to develop combination strategies to sensitize OC cells to platinum and PARP inhibitors.^11^, ^12^

Protein kinases catalyze protein phosphorylation and are involved in various cellular processes,^13^, ^14^ and their mutation or abnormal expression has been associated with carcinogenesis, metastasis, and chemoresistance.^15^, ^16^, ^17^ Several kinases have become important molecular targets and biomarkers for cancer.^18^, ^19^ CDC2‐like kinase (CLK) family, which consists of four isoforms: CLK1, CLK2, CLK3, and CLK4, is known for its bidirectional phosphorylation specificity for regions rich in serine‐arginine residues, and modulation of pre‐mRNA splicing.^20^, ^21^, ^22^ More and more studies link CLKs to cancer. It has been reported that CLK2 is highly expressed in various solid tumors (such as breast cancer, lung cancer, esophageal squamous cell carcinoma, and colorectal cancer), and acts as an oncogene.^23^, ^24^, ^25^, ^26^ Targeting CLK with inhibitors exhibit potent anticancer effects.^22^, ^27^ For example, a study indicated CLK2 as an oncogenic kinase in breast cancer, and the inhibition of CLK2 led to the upregulation of genes related to epithelial‐to‐mesenchymal transition (EMT).^23^ Nevertheless, the role of CLK in OC remains largely unknown.

Using gene microarray, we found that CLK2 expression was increased in OC tissues compared with the normal epithelial tissues, and a higher level of CLK2 was associated with platinum resistance and poor prognosis in OC. To date, the function of CLK2 in OC and whether CLK2 regulates drug resistance have not been reported. Therefore, we further demonstrated that silencing CLK2 sensitized OC cells to platinum treatment in vitro and in vivo. Mechanistically, CLK2 enhanced DNA damage repair and promoted chemoresistance by activating BRCA1. Targeting CLK2 in combination with PARP inhibitor is synergistic in inducing more severe DNA damage in cisplatin‐resistant OC cells, attenuating tumor growth, and prolonging survival time in mice bearing patient‐derived xenografts (PDXs). Moreover, we identified for the first time that p38 regulated CLK2 protein stability by specifically phosphorylating its threonine 343 (Thr343) site. Collectively, our data suggest that CLK2 may serve as a potential target for overcoming platinum resistance in OC patients, as well as providing a novel theoretical basis for individualized and precise treatment.

## 2 RESULTS

### 2.1 CLK2 is associated with platinum resistance and poor prognosis in OC patients

**FIGURE 1:**
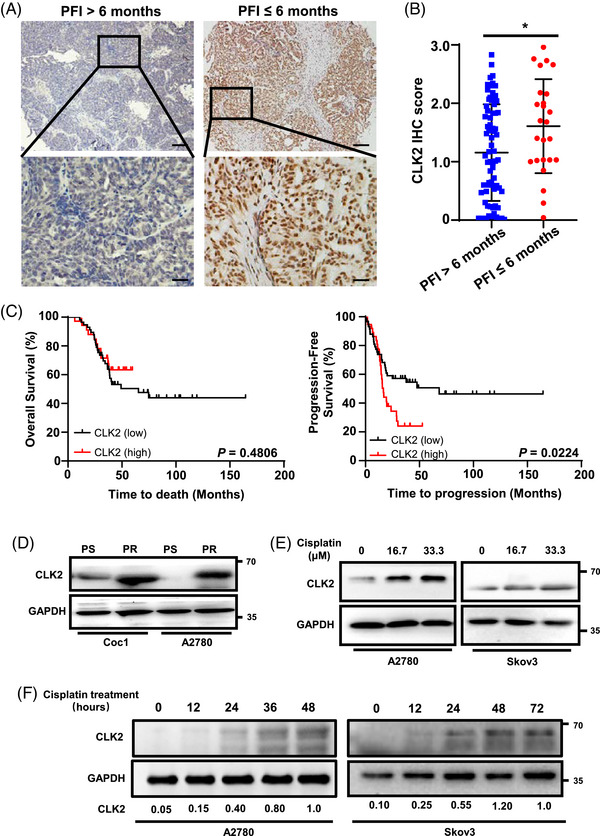
Cdc2‐like kinase 2 (CLK2) is associates with platinum resistance and poor prognosis in ovarian cancer patients. (A) Representative immunohistochemical images of CLK2 expression in ovarian cancer tissues. The level of CLK2 was scored according to the *H*‐score for CLK2. Scale bar for the images at ×10, 100 µm; scale bar for the images at ×40, 25 µm. (B) Comparison of CLK2 expression between patients with platinum‐free interval (PFI) > 6 months (blue) and PFI ≤ 6 months (red). *p* Values were determined by Student's *t*‐test. (C) Kaplan–Meier analysis of the overall survival (left panel) and progression‐free survival (right panel) of ovarian cancer patients with high (red) or low (black) CLK2 expression. The cutoff value between high and low groups was determined by the receiver operating characteristic (ROC) curve. *p* Values were determined by log‐rank test. (D) Western blot analysis of CLK2 expression in platinum‐resistant Coc1 (Coc1‐PR), platinum‐resistant A2780 (A2780‐PR) cells and their parental Coc1 and A2780 cell lines. CLK2 was upregulated in the PR cells compared with their platinum‐sensitive (PS) cells. (E and F) Western blot analyses of CLK2 expression in ovarian cancer cells treated with cisplatin. CLK2 expression was accumulated upon cisplatin treatment in a dose‐ (E) and time‐dependent manner (F). The A2780 and Skov3 cell lines were treated with cisplatin at different concentrations (0–33.3 µM) for 24 h, or with 33.3 µM cisplatin for different time intervals (0–72 h). GAPDH was used as the internal control. *p < 0.05.

Gene expression microarrays showed that CLK2 expression was elevated in OC tissues compared with normal ovarian tissues (Figure S1). To determine CLK2's clinical significance in OC, we performed immunohistochemical (IHC) staining for CLK2 across 97 OC tissues and summarized patients’ clinicopathological characteristics in Table S1. We next divided these OC tissues into low CLK2 expression (*n* = 60) and high CLK2 expression groups (*n* = 37) based on the receiver operating characteristic (ROC) curve of CLK2 expression. Analysis of the relationship between CLK2 protein levels and clinicopathological parameters indicated positive correlations between high CLK2 expression and short PFI (*p* = 0.033) as well as high risk of recurrence (*p* = 0.030) (Table S2). PFI is defined as the interval between the time of completion of platinum‐based chemotherapy and the date of first relapse or death.^8^ Generally, OC patients with a PFI of ≤6 months are typically considered as platinum resistant, and >6 months as platinum sensitive. Notably, patients with PFI ≤6 months exhibited higher CLK2 levels than those with PFI > 6 months (*p* < 0.05) (Figures 1A and B), indicating CLK2's potential involvement in platinum resistance. Kaplan‐Meier survival analysis and log‐rank testing further demonstrated that although the overall survival (OS) of OC patients with different CLK2 expressions had no significance (*p* = 0.4806), patients with higher CLK2 level had poorer progression‐free survival (PFS) (*p* = 0.0224) (Figure 1C). Univariate and multivariate analyses indicated that a high CLK2 level was an independent prognostic factor for the PFS (*p* = 0.009) of OC patients (Table S3). Collectively, these data suggest that CLK2 upregulation may be associated with platinum resistance and serve as a biomarker for the survival of OC patients.

Further, we examined CLK2 expression in OC cell lines and their associated PR cell lines. PR cell lines Coc1 (Coc1‐PR) and A2780 (A2780‐PR) were induced from their parental cells by continuous exposure to gradually increased concentrations of cisplatin. Western blotting showed that CLK2 expression was increased in the acquired PR cells compared with their parental cells (Figure 1D). Consistently, platinum treatment significantly increased CLK2 protein level in a dose‐ and time‐dependent manner in the parental cells (Figures 1E and F). These findings further support the hypothesis that CLK2 is involved in the development of acquired platinum resistance.

### 2.2 CLK2 retains platinum resistance by activating BRCA1 to enhance DNA damage repair

**FIGURE 2:**
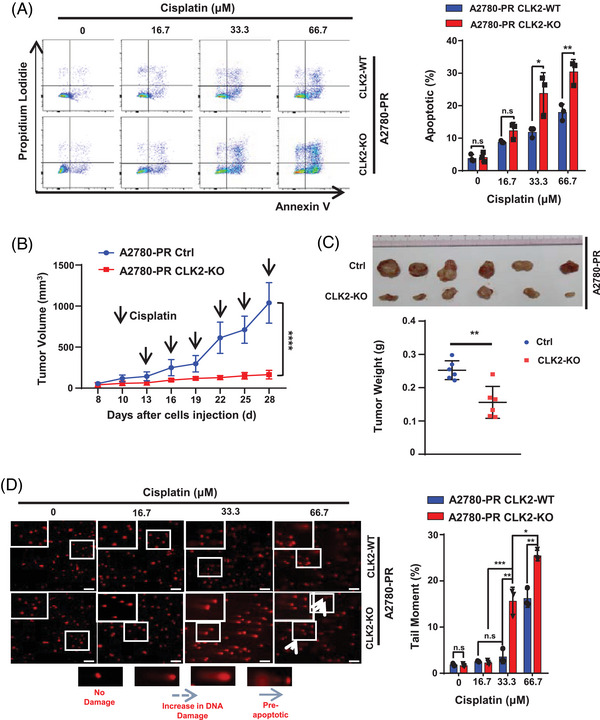
CLK2 retains platinum resistance by enhancing DNA damage repair. (A) A2780‐PR CLK2‐WT and A2780‐PR CLK2‐KO cells were treated with various concentrations of cisplatin for 24 h. Cell apoptosis was determined via flow cytometry using an Annexin‐V/PI dual staining assay (left panel). Columns (right panel) represented the average percentage of Annexin V‐positive, PI‐negative cells from three independent experiments, shown as the mean ± SD. *p* Values were determined by Student's *t*‐test. (B) Tumor volumes of nude mice inoculated with 1 × 106 A2780‐PR Ctrl (blue) or A2780‐PR CLK2‐KO (red) cells were monitored and recorded every 3 days since 8 days after inoculation (*n* = 6). Two weeks after inoculation, mice were administrated (i.p.) with cisplatin (5 mg/kg). Data were shown as mean ± SD. *p* Value was determined by Student's *t*‐test. (C) Representative image (top panel) and quantification of tumor weights (bottom panel) of nude mice inoculated with 1 × 106 A2780‐PR Ctrl (blue) or A2780‐PR CLK2‐KO (red) cells (*n* = 6). Tumor weights were measured on day 28. Data were shown as mean ± SD. *p* Values were determined by Student's *t*‐test. (D) CLK2 knockout decreased the DNA damage repair in the platinum‐resistant ovarian cancer cell. Representative images of the comet assay used to evaluate DNA damage of A2780‐PR CLK2‐WT and A2780‐PR CLK2‐KO cells after treatment with different concentrations of cisplatin (left panel). Columns represented the average percentage of tail moment in each group (right panel). *p* Values were determined by Student's *t*‐test. Scale bar for the images at ×10, 100 µm. n.s. no significance, *p < 0.05, **p < 0.01, ***p < 0.001, ****p < 0.0001.

To experimentally test the function of CLK2 in platinum resistance, we knocked out endogenous *CLK2* in A2780‐PR cells using the CRISPR‐Cas9 system (Figure S2). Compared with CLK2‐wide type (CLK2‐WT) clones, CLK2‐knocked out (CLK2‐KO) clones displayed significantly more apoptosis in both medium (33.3 µM, *p* = 0.033) and high (66.7 µM, *p* = 0.0084) platinum concentrations (Figure 2A), indicating CLK2 was necessary for retaining platinum resistance. To further confirm the role of CLK2 in platinum resistance in vivo, we performed a xenograft growth assay using A2780‐PR and A2780‐PR CLK2‐KO cells. As shown, the endpoint growth and weight of the CLK2‐KO xenograft tumors were significantly inhibited by cisplatin (Figures 2B and C). In summary, these in vitro and in vivo data demonstrated that silencing *CLK2* reduced platinum resistance of OC cells.

The therapeutic mechanism of platinum is to induce DNA damage and consequently cell death.^28^ Enhancing DNA damage repair is one way by which cells can become drug resistant.^28^, ^29^ We thus performed an alkaline comet assay to determine whether CLK2 influenced DNA damage repair. Cisplatin treatment induced a concentration‐dependent increase in DNA damage in both A2780‐PR WT and A2780‐PR CLK2‐KO cells. However, in both the medium (*p* = 0.0032) and high (*p* = 0.0021) cisplatin concentration of treatment groups, the amount of DNA tail was significantly higher in A2780‐PR CLK2‐KO cells than in A2780‐PR WT cells (Figure 2D), indicating that CLK2 knockout (KO) inhibited DNA damage repair in the PR OC cells.

**FIGURE 3:**
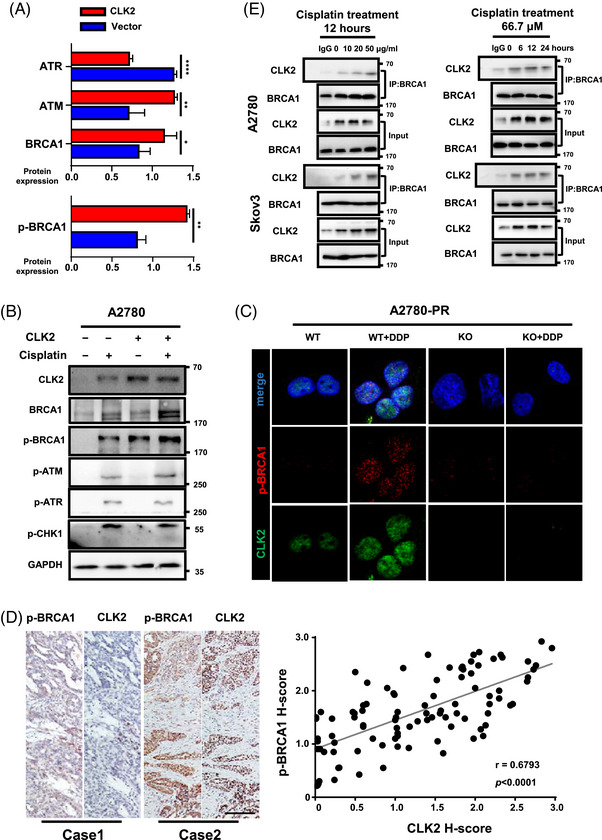
CLK2 binds to and activates breast cancer gene 1 (BRCA1) to enhance DNA damage repair. (A) Proteins altered under the overexpression of CLK2 in A2780 cells through proteome and phosphoproteome analyses. *p* Values were determined by Student's *t*‐test. (B) Western blot analysis of the protein levels of DNA damage repair related proteins in A2780 cells transfected with vector or CLK2 plasmids in the presence or absence of cisplatin (16.7 µM, 12 h). The overexpression of CLK2 increased the expression of p‐BRCA1. (C) Representative immunofluorescence images of CLK2 (green), p‐BRCA1 (red), and DAPI (blue) in A2780‐PR CLK2‐WT and A2780‐PR CLK2‐KO cells in the presence or absence of cisplatin for 24 h. Scale bar for the images at ×100, 10 µm. (D) Correlation analysis of CLK2 and p‐BRCA1 expressions in ovarian cancer tissues (*n* = 97). Representative immunohistochemical images of CLK2 and p‐BRCA1 in ovarian cancer tissues were shown (left panel). CLK2 protein level was positively correlated with the protein level of p‐BRCA1 (right panel). Scale bar for the images at ×10, 100 µm. (E) Coimmunoprecipitation assays showing the presence of a complex containing CLK2 and BRCA1. Cells were treated with different concentrations of cisplatin for 12 h (left panel) or with 66.7 µM cisplatin for different hours (right panel). Input, protein expression in cell lysates detected by Western blot. IgG, negative control. IP, expression of compound coprecipitated by BRCA1 antibody. *p < 0.05, **p < 0.01, ****p < 0.0001.

We further investigated how CLK2 enhanced DNA damage repair. First, we detected whether CLK2 regulated DNA damage repair proteins through proteome and phosphoproteome analyses. Among the candidates, *BRCA1* was the most significantly increased gene in the CLK2 overexpressing cells compared with the vector cells (Figure 3A). Noting that BRCA1 is a critical tumor suppressor enzyme for DNA double‐strand break (DSB) repair,^30^, ^31^ we selected BRCA1 as our investigation focus. To demonstrate BRCA1 as a downstream mediator of CLK2, we knocked down *BRCA1* in CLK2 overexpressing cells (Figure S3A). Cell surviving assays showed that compared with control group, CLK2 overexpression increased a 2.68‐fold in IC_50_ of cisplatin, which was suppressed by inhibiting BRCA1 (Figure S3B). It is reported that BRCA1 is phosphorylated by various kinases, including the ataxia‐telangiectasia mutated (ATM) kinase, ATM‐related kinase (ATR) and checkpoint kinase 1 (CHK1) upon DNA damage.^32^, ^33^ The phosphorylation sites of BRCA1 in response to DNA damage, such as Ser1423 and Ser1524, have also been reported.^34^, ^35^ Herein, we found that overexpression of CLK2 activated p‐BRCA1 and increased expression of p‐BRCA1, but did not alter the phosphorylated levels of ATM, ATR, and CHK1 (Figure 3B). Moreover, the p‐BRCA1 level in cisplatin‐treated cells was remarkably higher than the control group, and when *CLK2* was knocked out, the cisplatin‐induced p‐BRCA1 expression was abolished significantly (Figure 3C), indicating that cisplatin increased p‐BRCA1 level via CLK2. Consistently, analysis of clinical samples demonstrated a positive correlation between CLK2 and p‐BRCA1 protein levels (Figure 3D). Notably, we performed a coimmunoprecipitation (Co‐IP) assay in OC cells and found that the abundance of BRCA1‐binded CLK2 was increased upon cisplatin treatment (Figure 3E).

Taken together, these results proposed that CLK2‐induced DNA damage repair can be attributed to the overexpression and activation of BRCA1.

### 2.3 p38 phosphorylates and stabilizes CLK2 at Thr343 after cisplatin treatment

**FIGURE 4:**
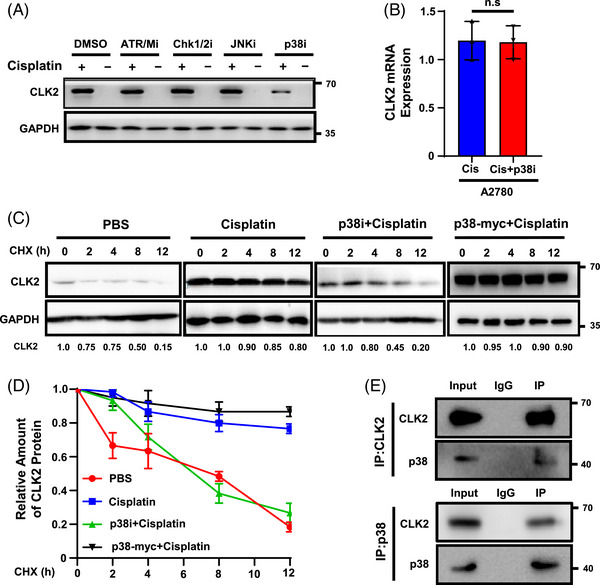
Cisplatin induces CLK2 upregulation via p38. (A) Western blot analysis of CLK2 expression in A2780 cells treated with various signaling pathway inhibitors or dimethyl sulfoxide (DMSO) in the presence or absence of cisplatin. Only p38 inhibitor attenuated the expression of CLK2. (B) CLK2 mRNA level in A2780 cells treated with cisplatin in the presence or absence of p38 inhibitor was measured by qRT‐PCR. Data was shown as mean ± SD. *p* Values were determined by Student's *t*‐test. (C) A2780 cells treated with phosphate‐buffered saline (PBS), or cisplatin, or cisplatin plus p38 inhibitor (p38i), or cisplatin and p38 overexpression (p38‐Myc) were incubated with 20 µg/mL cycloheximide (CHX) for the indicated periods and then analyzed by Western blot. The results were normalized to the levels of GAPDH. (D) Quantitation of CLK2 protein levels was based on the Western blot result. Data were shown as mean ± SD. (E) Coimmunoprecipitation assay showing the presence of a complex containing CLK2 and p38. CLK2 antibody coprecipitating p38 (top panel). p38 antibody coprecipitating CLK2 (bottom panel). Input, protein expression in cell lysates detected by Western blot. IgG, negative control. IP, expression of compound coprecipitated by CLK2 or p38 antibody. n.s., no significance.

We next investigated the underlying mechanism between platinum stimulation and CLK2 upregulation. First, we found that the mRNA expression of CLK2 did not upregulate when treated with cisplatin (Figure S4). To identify the molecule that activates CLK2 upon cisplatin treatment, we examined CLK2 expression after treatment with a variety of kinases inhibitors that could be activated by cisplatin,^36^, ^37^, ^38^ including AZD6738 (ATR inhibitor), KU‐60019 (ATM inhibitor), AZD7762 (CHK1/CHK2 inhibitor), SP600125 (Jun‐N‐terminal kinase inhibitor), and VX702 (p38α mitogen‐activated protein kinase [MAPK] inhibitor). Results showed that only the p38 inhibitor attenuated the effect of cisplatin in increasing the CLK2 protein level (Figure 4A). Interestingly, the VX702 treatment did not alter CLK2 mRNA expression, indicating the effect of p38 on CLK2 was not via direct transcriptional regulation (Figure 4B). Next, we used cycloheximide (CHX), a protein translation inhibitor, to explore whether p38 could increase CLK2 protein stability. Results showed that under the treatment of cisplatin, the protein stability of CLK2 was enhanced. When p38 was inhibited, CLK2 destabilized substantially by about 50% after 8 h, indicating that its cisplatin‐enhanced stability could be blocked by p38 inhibitor. Conversely, overexpression of p38 promoted the enhancement of cisplatin on the CLK2 protein level (Figures 4C and D). To further explore the relationship of p38 and CLK2, we examined the interaction between p38 and CLK2 by Co‐IP assay. A clear interaction was observed between CLK2 and p38 in A2780 cells (Figure 4E). These findings indicate that cisplatin induces and stabilizes CLK2 protein via the p38 pathway.

**FIGURE 5:**
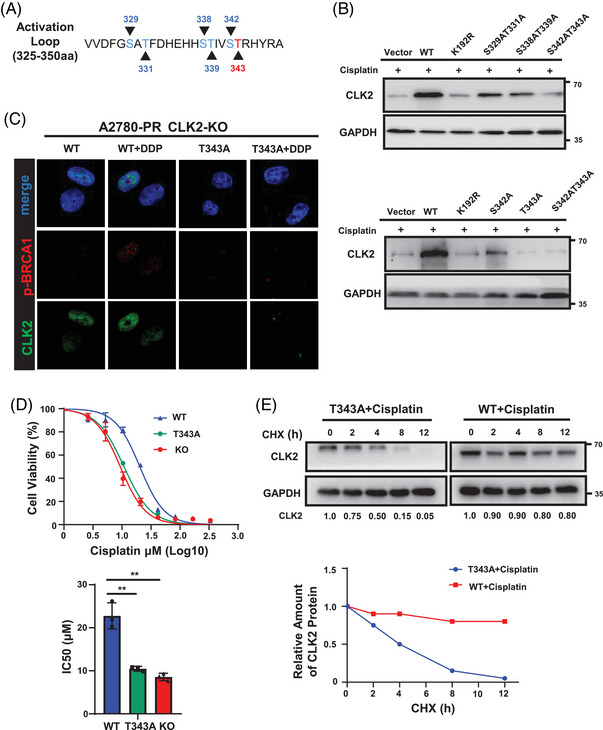
p38 phosphorylates CLK2 at threonine 343 after cisplatin treatment. (A) The schematic diagram showed the CLK2 activation loop with putative p38 phosphorylation sites. Amino acid sites S329, T331, S338, T339, S342, T343 were labeled with arrows. (B) Western blot analyses of CLK2 expression in A2780‐PR CLK2‐KO cells transfected with CLK2‐WT or its mutant plasmids. Cells were treated with cisplatin (33.3 µM) 24 h after transfection. (C) Representative immunofluorescence images of CLK2 (green), p‐BRCA1 (red), and DAPI (blue) in A2780‐PR CLK2‐KO cells transfected with CLK2‐T343A plasmid. 24 h after transfection, cells were treated with cisplatin (33.3 µM) for another 24 h. Scale bar for the images at ×100, 10 µm. (D) CCK8 assays of A2780‐PR CLK2‐KO cells transfected with CLK2‐WT (blue), CLK2‐T343A (green), or vector (red) plasmids. Cells were treated with cisplatin under a range of concentrations as indicated (top panel). IC_50_ values for cisplatin were calculated using nonlinear regression and shown as mean ± SD (bottom panel). *p* Values were determined by Student's *t*‐test. (E) A2780 cells transfected with CLK2‐T343A or CLK2‐WT plasmids for 36 h were incubated with 20 µg/ml CHX for the indicated periods. 24 h after transfection, cells were treated with 33.3 µM cisplatin for 24 h and then analyzed by Western blot. The results were normalized to the levels of GAPDH (top panel). Quantitation of CLK2 protein levels was based on the Western blot result (bottom panel). **p < 0.01.

We next explored how cisplatin stabilized CLK2 proteins via p38. It has been reported that phosphorylation of CLK2 leads to its overexpression.^39^ As a kinase family, CLK activation loops are evolutionarily conserved, and their key amino acid positions are likely to be in the 320−350th amino acid sequence range.^39^, ^40^, ^41^ Therefore, we inferred that CLK2's potential phosphorylation sites were in this sequence. To identify the exact phosphorylation sites, we generated several *CLK2* point mutants in which Ser329, Thr331, Ser338, Thr339, Ser342, and Thr343 were converted to alanine (S329A, T331A, S338A, T339A, S342A, and T343A) (Figure 5A). We transfected A2780‐PR CLK2‐KO cells with vector, CLK2‐WT and CLK2‐mutants. K192R, which is a kinase‐inactive mutant of CLK2,^42^ was also transfected as a positive control. Results showed that in the presence of cisplatin, CLK2 protein level was only diminished in cells transfected with the T343A plasmid (Figure 5B), suggesting that it was the site Thr343 that most significantly affected CLK2 activity and expression in response to cisplatin treatment. Cell immunofluorescence further showed that when the Thr343 site was mutated, CLK2 expression was attenuated, resulting in p‐BRCA1's inability to be activated even after platinum treatment (Figure 5C). Furthermore, cell surviving assay demonstrated that mutation of Thr343 in A2780 restored sensitivity to cisplatin (Figure 5D). In addition, the half‐life of T343A‐CLK2 was significantly short in A2780 cells with cisplatin treatment (Figure 5E). These findings indicate that the Thr343 site of CLK2 is a novel target site to interact with p38 in response to cisplatin treatment.

### 2.4 p38 and ring finger protein 8 mediate CLK2 protein ubiquitination

**FIGURE 6:**
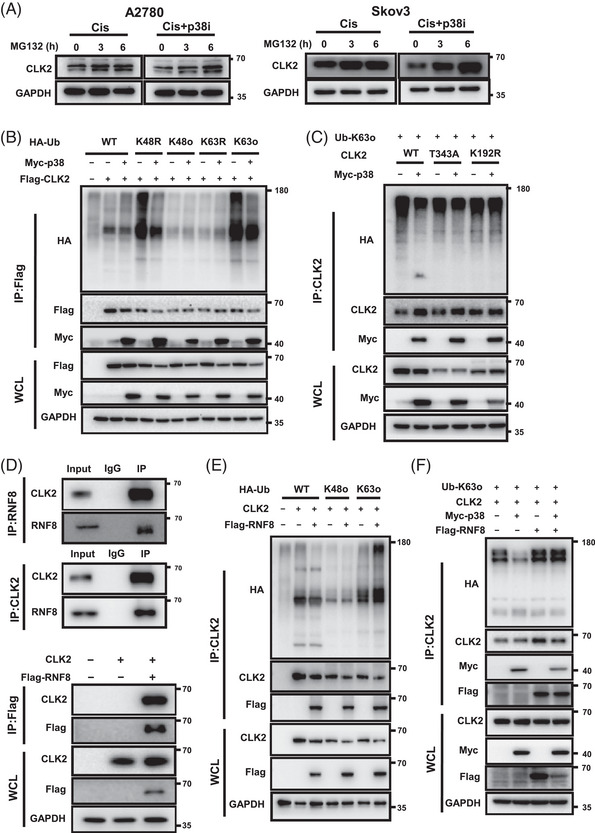
p38 and ring finger protein 8 (RNF8) mediate CLK2 protein ubiquitination. (A) Western blot analyses of CLK2 expression in A2780 and Skov3 cells which were treated with cisplatin (16.7 µM) in the presence or absence of p38 inhibitor (10 µM). Cells were incubated with MG132 (10 µM) for the indicated periods. (B) Ubiquitination assays of CLK2 in human embryonic kidney (HEK293T) cells transfected with Flag‐CLK2, Myc‐p38, and HA‐ubiquitin or its mutants (K48R, K48o, K63R, K63o) plasmids. IP, expression of compound coprecipitated by Flag antibody. WCL, whole cell lysate. (C) Ubiquitination assays of CLK2 in HEK293T cells transfected with Myc‐p38, HA‐Ub‐K63o, and CLK2‐WT or its mutants (T343A, K192R) plasmids. IP, expression of compound coprecipitated by CLK2 antibody. WCL, whole cell lysate. (D) Coimmunoprecipitation assay showing the endogenous (top panel) presence of a complex containing CLK2 and RNF8 and the exogenous (bottom panel) presence of a complex containing CLK2 and Flag‐RNF8. Input, protein expression in cell lysates detected by Western blot. IgG, negative control. IP, expression of compound coprecipitated by CLK2, RNF8, or Flag antibody. WCL, whole cell lysate. (E) Ubiquitination assays of CLK2 in HEK293T cells transfected with CLK2, Flag‐RNF8, and HA‐ubiquitin or its mutants (K48o, K63o) plasmids. IP, expression of compound coprecipitated by CLK2 antibody. WCL, whole cell lysate. (F) Ubiquitination assays of CLK2 in HEK293T cells cotransfected by various combinations of plasmids expressing CLK2, Myc‐p38, Flag‐RNF8, and HA‐Ub‐K63o. IP, expression of compound coprecipitated by CLK2 antibody; WCL, whole cell lysate.

Ubiquitination is the most widespread pathway for protein degradation. As a previous study has reported it as the pathway by which CLK2 undergoes degradation,^39^ we speculated whether p38 affected the ubiquitination of CLK2. We used MG132, a proteasome inhibitor, and observed that the effect of p38 inhibitor in reducing the CLK2 protein level was attenuated (Figure 6A). Then, we performed ubiquitination assays in human embryonic kidney (HEK293T) cells transfected with Myc‐p38, Flag‐CLK2 and HA‐ubiquitin WT, or mutation plasmids (K48R, K48o, K63R, or K63o mutants) to identify the lysine residue site of ubiquitin modification. As shown in Figure 6B, overexpressed p38 in HEK293T cells inhibited the poly‐ubiquitination of CLK2. Interestingly, the inhibited CLK2 ubiquitination by p38 was mainly extended through the K63‐linkage but not the K48‐linkage. However, after mutating the T343 site of CLK2, its ubiquitination level was no longer affected by p38 overexpression (Figure 6C). These data show that p38 inhibits the K63‐linked poly‐ubiquitination of CLK2 by binding to its T343 site, and thus stabilizes its expression.

We further aimed to elucidate which E3 ubiquitin kinase participates in the p38‐suppressed CLK2 ubiquitination. We screened for potential CLK2 binding proteins (https://thebiogrid.org/), and focused on ring finger protein 8 (RNF8), a RING domain well‐known E3 ubiquitin ligase. As shown, the exogenous and endogenous interactions between RNF8 and CLK2 were observed (Figure 6D). Notably, the ubiquitination of CLK2 induced by RNF8 was detected at the K63‐linkage (Figure 6E). Ubiquitination assays in HEK293T cells transfected with CLK2, Myc‐p38, Flag‐RNF8, and HA‐Ub‐K63o showed that inhibition of p38‐induced CLK2 ubiquitination could be rescued by upregulating RNF8 (Figure 6F), indicating that RNF8 may mediate p38‐suppressed CLK2 ubiquitination.

### 2.5 Combination of CLK2 and PARP inhibitors achieves a “synergistic lethal effect”

**FIGURE 7:**
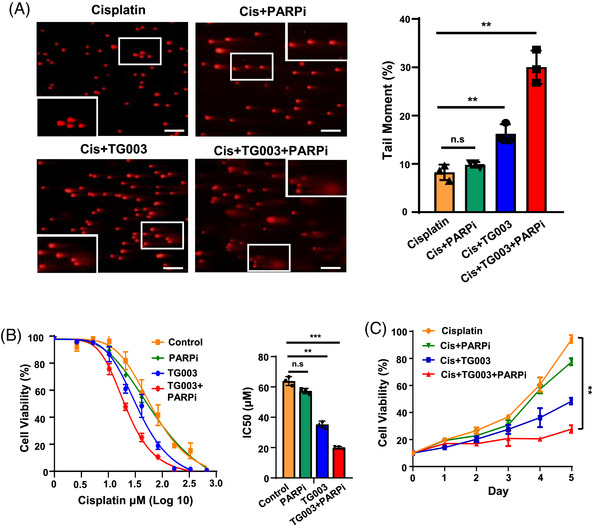
Combination of CLK2 and poly ADP‐ribose polymerase (PARP) inhibitors achieves a “synergistic lethal effect” in ovarian cancer cells. (A) The combination of CLK2 and PARP inhibitors increased DNA damage in A2780‐PR cells. Representative images of the comet assay used to evaluate DNA damage in A2780‐PR cells treated with cisplatin (16.7 µM) alone (yellow) or together with PARPi (green), TG003 (blue), or TG003 plus PARPi (red) for 24 h, respectively (left panel). Columns represented the average percentage of tail moment in four groups (right panel). *p* Values were determined by Student's *t*‐test. Scale bar for the images at ×10, 100 µm. (B) CCK8 assays of A2780‐PR cells treated with PBS (yellow), or PARPi (green), or TG003 (blue), or TG003 plus PARPi (red) for 24 h, respectively. Cells were treated with cisplatin under a range of concentrations as indicated (left panel). IC_50_ values for cisplatin were calculated using nonlinear regression and shown as mean ± SD (right panel). *p* Values were determined by Student's *t*‐test. (C) Cell viability analysis of A2780‐PR cells treated with cisplatin (yellow) alone, or together with PARPi (green), TG003 (blue), or TG003 plus PARPi (red) for 5 days. *p* Values were determined by Student's *t*‐test. n.s., no significance; **p < 0.01, ***p < 0.001.

The above results indicated that a lack of CLK2 leads to decreased DNA damage repair via inhibiting phosphorylated activation of BRCA1, thereby simulating a “BRCAness” status.^43^, ^44^ In OC patients with BRCA mutations, the PARP inhibitor, like Olaparib, has been proven to have an effect on increasing DNA DSBs and decreasing cell viability.^45^ We hypothesized that combining CLK2 and PARP inhibition could achieve a synergistic lethal effect. As shown in Figure 7A, treatment with Olaparib only marginally increased the amount of DNA in the tail compared with the baseline levels, suggesting that the PARP inhibitor alone was insufficient to overcome platinum resistance. Meanwhile, treatment with the CLK2 inhibitor, TG003, increased DNA damage with less than 10% DNA in the tail compared with control cells. Notably, the combination of PARP and CLK2 inhibitors effectively induced more than 20% DNA damage in the tail compared with the control group. Consistently, dose–response curves showed that the combination of PARP and CLK2 inhibitors was more effectively synergistic in decreasing the IC_50_ of A2780‐PR than any single agent (Figure 7B). Furthermore, coadministration of PARP and CLK2 inhibitors resulted in more potent inhibition of the proliferation of PR cells exposed to cisplatin than any single‐agent treatment (Figure 7C).

**FIGURE 8:**
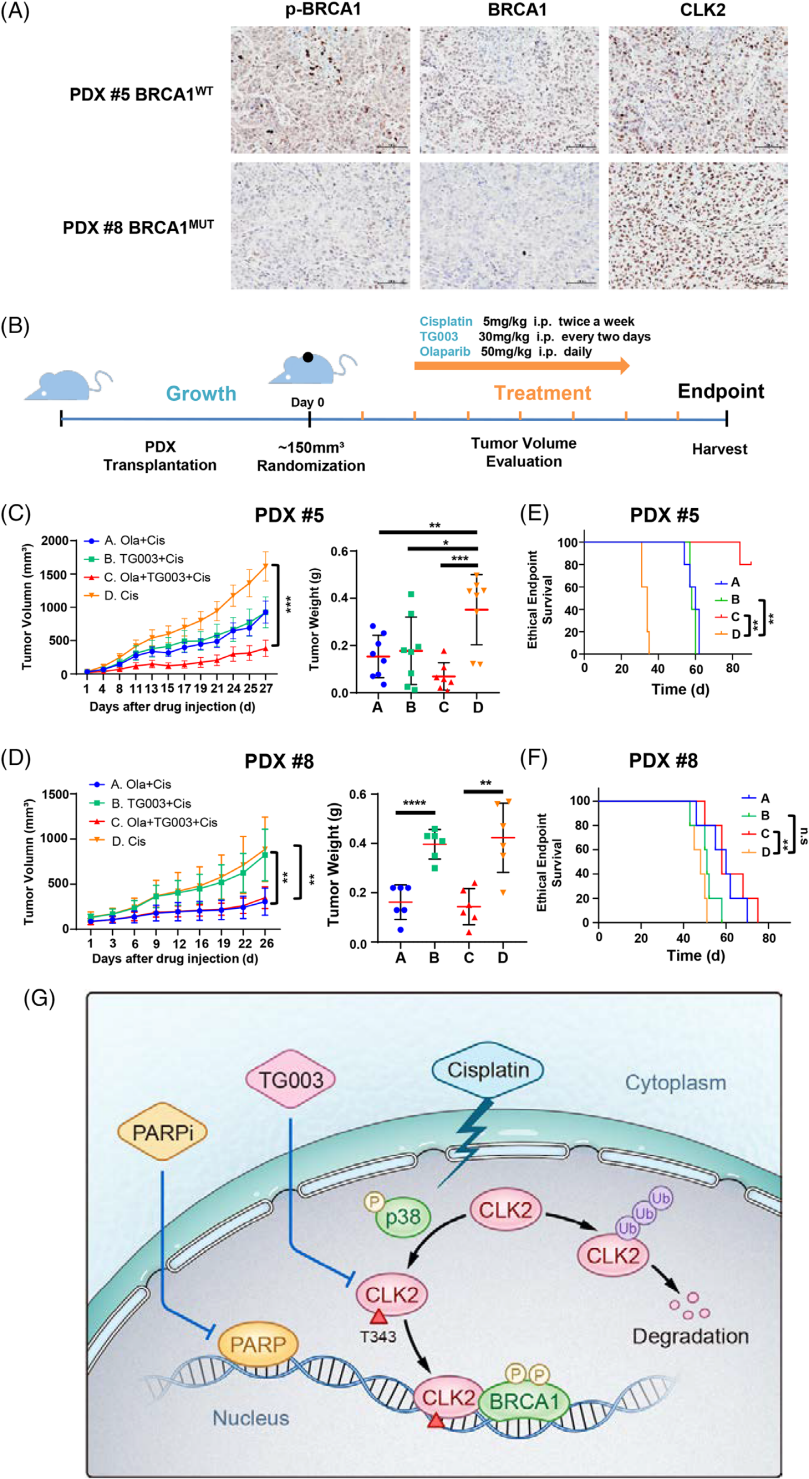
Combination of CLK2 and PARP inhibitors achieves a “synergistic lethal effect” in patient‐derived xenografts (PDX) models. (A) Representative immunohistochemical images of CLK2, BRCA1 and p‐BRCA1 in tissues from ovarian cancer patients #5 and #8. Scale bar for the images at ×20, 100 µm. (B) Treatment scheme of mice bearing PDX. (C and D) Nude mice bearing #5 (C, *n* = 8 per group) and #8 (D, *n* = 6 per group) PDX tumors were divided into four groups randomly after the tumor volumes reached 150 mm^3^ and were treated with cisplatin (yellow) alone, or together with Olaparib (blue), TG003 (green), or Olaparib plus TG003 (red), respectively. The tumor volumes were monitored and recorded (left panel). Tumors were weighted after tumor excision (right panel). Error bars indicate SD. *p* Values were determined by Student's *t*‐test. (E and F) Kaplan–Meier survival curves of #5 (E) and #8 (F) PDX tumor‐bearing mice (*n* = 5 per group). Drugs were injected intraperitoneally when the tumor volume reached 150 mm^3^ approximately. *p* Values were determined by log‐rank test. (G) Schematic diagram describing that targeting the CLK2/BRCA1 axis would overcome platinum resistance in ovarian cancer. *p < 0.05, **p < 0.01, ***p < 0.001, ****p < 0.0001.

To examine the synergistic efficacy of CLK2 and PARP inhibition for increasing chemosensitivity in vivo, we utilized PDX models, which recapitulate patient tumor heterogeneity and histopathology and have been used to predict the clinical response to chemotherapy. As shown in Figure 8A, two patients exhibited distinct statuses of CLK2, BRCA1, and p‐BRCA1. Therefore, we adopted these two PDX models to analyze the efficacy of different treatments (Figure 8B).

In PDX #5, TG003 or Olaparib inhibited the growth of tumors compared with the vehicle group, and the combination of TG003 and Olaparib achieved a more effective inhibition effect (Figure 8C). However, in PDX #8, TG003 failed to increase chemosensitivity (Figure 8D). Furthermore, we divided another 20 tumor‐bearing mice into four groups to access the effect on survival, and the ethical endpoint was determined when tumor volume ≥1500 mm^3^. We observed that in PDX #5, the combination of TG003 and Olaparib prolonged survival compared with other three groups (Figure 8E). In PDX #8, TG003 failed to prolong survival compared with the vehicle group (Figure 8F). This is consistent with our hypothesis that a normal BRCA1 function is required for CLK2‐promoted chemoresistance. No signs of tissue damage were detected in the liver, kidney, lung or pancreas of tumor‐bearing mice (Figure S5). Treatment with cisplatin had a limited impact on bodyweight during tumor regression (Figure S6). Collectively, these findings reveal that pharmacological inhibition of PARP alone sensitizes the tumors to platinum‐based chemotherapy, while its combination with CLK2 inhibitor contributes to a more powerful effect of platinum in the WT group. Altogether, these data might support the synergistic effect of PARP‐CLK2 inhibition in BRCA1^WT^ cisplatin‐resistant OC patients.

## 3 DISCUSSION

This study identified a previously unknown regulatory mechanism that p38 phosphorylates Thr343 of CLK2 to increase CLK2 protein stability by blocking its ubiquitination and degradation. Increased CLK2 activates BRCA1 to enhance DNA damage repair and induced resistance to cisplatin. Both in vitro and in vivo data demonstrated that inhibition of CLK2 reversed platinum resistance of OC cells, and, when combined with a PARP inhibitor, synergistically suppressed tumor growth in the BRCA1^WT^ PDX model but not the BRCA1^MUT^ PDX model. Our results indicate that the PARP‐CLK2 dual inhibitors combination may serve as a promising therapeutic strategy for PR OC patients, especially BRCA1^WT^ patients (Figure 8G).

To our knowledge, this is the first time to report that CLK2 is able to promote platinum resistance. Recently, CLK2 is known for its function in pre‐mRNA splicing.^20^, ^21^, ^22^, ^23^, ^27^ In luminal breast cancer cells, CLK2 modulates the splicing of mRNAs in the EMT pathway to regulate tumor development.^23^ In this work, we discovered a new mechanism of CLK2, which was wholly different from its function in pre‐mRNA splicing. Herein, we found that CLK2 was increased in PR OC cells and associated with poor survival of OC patients. Both in vivo and in vitro experiments confirmed that CLK2 promoted platinum resistance by activating BRCA1 and enhancing DNA damage repair. Our study reveals additional diversity in CLK2 functions and provides novel ideas for subsequent exploration of CLK2.

Platinum‐based drugs kill tumor cells by causing DNA cross‐link breaks, including single‐strand breaks (SSB) and, more deadly, DSBs.^28^, ^29^, ^46^, ^47^ SSB can be repaired by PARP, which recruits downstream proteins to participate in the DNA repair process.^46^ In DSB, homologous recombination repair is the most accurate repair method, and BRCA1 plays an essential role in this process.^47^ Theoretically, OC patients with BRCA1^MUT^ display abnormal function in DSB repair, and PARP inhibitors exploit this vulnerability to induce tumor cell death eventually.^45^, ^48^, ^49^ Indeed, extensive clinical research has shown that OC patients who harbor BRCA1^MUT^ benefit the most from PARP inhibitor.^49^ However, BRCA^WT^ patients account for almost 80% of OC cases, and how to improve the treatment efficacy for these patients is a critical issue. In our study, inhibiting CLK2 results in defective BRCA1 function, which effectively causes a “BRCAness” status. Therefore, the combined use of PARP and CLK2 inhibitors provides an approach to achieve a synergistic lethal effect in BRCA1^WT^ OC cells and PDX models. Notably, these findings provide an entirely new and effective strategy for most OC patients with BRCA1^WT^ and poor efficacy of PARP inhibitor. Moreover, it has been reported that the spontaneous reversion of BRCA mutation that restores the open reading frame represents a possible pathway of platinum resistance in BRCA^MUT^ OC patients.^50^, ^51^, ^52^ In these patients, if the state of BRCAness could be achieved, their tumor cells would be resensitized to chemotherapy, indicating that the CLK2 inhibitor may be also effective. Taken as a whole, we suggest a novel theoretical basis for individualized and precise treatment of OC patients based on CLK2 inhibitor.

Another novelty in our study is our identification of the previously unknown regulatory mechanism that p38 is able to phosphorylate Thr343 of CLK2 to increase CLK2 protein stability. Nam et al.^53^ have reported that Ser34 and Thr127 residues of CLK2 are phosphorylated by Akt to increase cell proliferation and viability after ionizing irradiation. In this study, we discovered for the first time that p38 is another upstream molecule to phosphorylate CLK2. Moreover, the MAPK signaling pathway plays an important role in cell functions.^54^ To date, p38 has been a molecule of abundant study, and a number of drugs targeting p38 have been developed for clinical research in the past decades. Unfortunately, most of these targeted drugs failed to be clinically adopted due to their severe side effects.^55^, ^56^ In triple negative breast cancer, the combination of targeting p38 and PARP did not induce significant cell death compared with each drug individually.^57^ We identified CLK2 as a novel downstream molecule of p38, and the CLK2 inhibitor is currently under investigation for the futural clinical trials to treat degenerative diseases and cancers.^58^, ^59^ Therefore, CLK2 inhibitor may play a pivotal role in the treatment of OC as an alternative to p38 inhibitor to a certain extent, and it is worthful to test this hypothesis in the future.

There are also limitations in our research. First, as a downstream molecule of p38, CLK2 mediates the expression of BRCA1 and enhances platinum resistance in OC. It remains unelucidated whether CLK2 has other diverse functions in OC. In addition, whether CLK2 has a similar role in other cancers, remains to be studied. Second, the OS of different CLK2 expression had no significance. There were many factors influencing patients’ OS, and death reason included many nontumor‐associated disease or accidents. What is more, there were some patients received different kinds of therapy including immuno‐therapy, molecular target therapy, and so on, which might affect the result of OS. The limited number of patients also restrict the results. Therefore, a large number of patients should be included to enhance the correction of this study. Third, our results suggest that the combination of CLK2 and PARP inhibitors has a more lethal therapeutic effect in both PR OC cells and BRCA1^WT^ PDX models. A relevant clinical trial is still under application to be conducted, and a long‐term collection of scientific and prospective data will more convincingly demonstrate that the CLK2 inhibitor is beneficial to BRCA1^WT^ OC patients.

In conclusion, this work reveals a novel p38–CLK2–BRCA1 axis in inducing platinum resistance in OC. These findings contribute to the understanding of the underlying role of CLK2 in response to platinum treatment, and emphasize the potential application of a combination of PARP and CLK2 inhibitions as an individualized therapy for OC patients.

## 4 MATERIALS AND METHODS

### 4.1 Patients and samples

OC tissues were collected from patients who did not receive therapy prior to tumor debulking operation from Sun Yat‐sen University Cancer Center. All patients were high‐grade serous ovarian carcinoma, and their postoperative histopathological types were reviewed according to WHO criteria by two experienced gynecological pathologists.

### 4.2 Cell culture and treatment

HEK293T cells were obtained from American Type Culture Collection (Manassas, USA), and human OC cell lines A2780, Coc1, A2780‐PR, Coc1‐PR, Skov3 were obtained from Shanghai Bioresource Collection Center (Shanghai, China). A2780‐PR and Coc1‐PR cells were developed from the parental cells by sequential exposure to increasing concentrations of cisplatin. All cell lines were cultured in high‐glucose Dulbecco's modified Eagle's medium (DMEM; #C11995500BT; GIBCO) supplemented with 10% fetal bovine serum (FBS; #A3161002C; GIBCO) and 1% penicillin–streptomycin (#15070063; GIBCO) at 37°C with 5% CO_2_ in an incubator. All cell lines were authenticated using short‐tandem repeat profiling less than 6 months ago, when this project was initiated, and the cells were not cultured for more than 2 months.

### 4.3 RNA inference

Short interfering RNA (siRNA) were designated specifically against BRCA1 and were purchased from GenePharma Co., Ltd. (Suzhou, China). The sequences were summarized in Table S4. For transfection, 4 µg siRNA was dissolved in 125 µL of Optimem media (#31985070; GIBCO). In another tube, the transfection medium Lipofectamine 3000 (#L3000015; Invitrogen) was dissolved in 125 µL of Optimen media. The two mixtures were then combined and incubated for 20 min at room temperature (RT). The mixture was added to cells which was plated in a six‐well plate with 2 mL fresh media. 48 h after transfection, cells were harvested and the effect of gene silencing was examined by Western blot.

### 4.4 Plasmids, virus production, and infection

pReceiver‐Lv121‐CLK2 and CLK2‐T343A plasmids were obtained from GeneCopoeia Inc. (Guangzhou, China). HA‐ubiquitin (WT, K48R, K48o, K63R, K63o) plasmids were kindly provided by Prof. Yunfei Yuan from Sun Yat‐sen University Cancer Center. Flag‐CLK2, Myc‐p38, Flag‐RNF8 plasmids were purchased from WeiZhen Biosciences Inc. (Jinan, China).

To produce retrovirus, HEK293T cells were cotransfected with the pReceiver‐Lv121‐CLK2 plasmid and packaging plasmids (PLP1, PLP2, and PLP/VSVG, which were kindly provided by Prof. Xinyuan Guan from Sun Yat‐sen University Cancer Center) using Lipofectamine 3000. At 48 h posttransfection, the supernatants were collected and filtered using a 0.45‐µm cellulose acetate filter (#YA0673; Solarbio). Subsequently, cells were incubated in culture medium with supernatants containing virus particles and supplemented with 8 µg/mL polybrene (#TR‐1003‐G; MilliporeSigma) for 12 h, followed by replacement with fresh medium. At 48 h postinfection, puromycin (2 µg/mL, #P8833; MilliporeSigma) was added to screen the infected cells.

### 4.5 Generation of CLK2 KO cells

The CRISPR/Cas9 system was used to KO CLK2 in A2780 and A2780‐PR cells. Briefly, cells were transfected with CLK2 single‐guide RNA (sgRNA) plasmid. 48 h after transfection, cells were selected by G418 (400 µg/mL; #A2513; APExBIO), and were sorted into a 96‐well plate. Single‐cell clones were expanded and validated as CLK2 KO clone by Western blot.

The CLK2 sgRNA plasmid (#HCP001098‐CG01‐3‐10‐b) was purchased from GeneCopoeia Inc.. The sgRNA sequence targeting CLK2's exon was: 5′‐GGAAGGCCATGTGGCGCACT‐3′.

### 4.6 Cell apoptosis assay

Apoptosis assays were conducted using an Annexin V‐FITC/PI Apoptosis Detection Kit (#BB‐4101‐3; BestBio). Cells were treated with different concentrations of cisplatin (#A8321; APExBIO) for 24 h. Apoptotic cells were then collected by trypsin without EDTA (#BL526A; Biosharp), washed with phosphate‐buffered saline (PBS; #BL302A; Biosharp) thrice and stained with Annexin V‐FITC and propidium iodide (PI) according to the manufacturer's protocol. Early apoptotic cells (Annexin V‐positive, PI‐negative) and late apoptotic cells (Annexin V‐positive, PI‐positive) were determined by flow cytometry (BD Biosciences, San Diego, USA) and the results were analyzed using FlowJo 10 software (Tree Star, Ashland, USA).

### 4.7 Cell proliferation assay

A2780‐PR cells (1 × 10^3^ cells/well) were seeded into 96‐well plates (100 µL cell suspensions) and either treated with cisplatin (33.3 µM), cisplatin and Olaparib (23 µM, #A4154; APExBIO), cisplatin and TG003 (10 µM, #B1431; APExBIO), or cisplatin, Olaparib, and TG003. Cell number was assessed every 24 h by Cell Counting Kit‐8 (CCK8; #B34304; Bimake) assays according to the manufacturer's instructions.

### 4.8 Comet assay

Cells were harvested posttreatment and processed for alkaline comet assay using a comet assay kit (#KGA240; KeyGEN), according to the manufacturer's protocol. Briefly, cells were washed with 1 × PBS, suspended in low melting‐point agarose (1% in PBS) and plated onto the standard agarose‐coated slides. The slide was then immersed in the lysis buffer at 4°C for 1 h in the dark. Next, the slide was placed into an electrophoresis chamber with electrophoresis solution for 1 h, and then the electrophoresis was carried out for 30 min at 25 V. After electrophoresis, the slide was washed with neutralizing solution and stained with PI (5 µg/mL). Each slide was photographed under a Zeiss Axio Imager Z2 microscope (LSM 880; Carl Zeiss, Oberkochen, Germany), and the tail moment percentage was computed using the Comet Assay IV software (Perceptive Instruments Ltd., Cambridge, UK). At least two hundred cells in each group were analyzed.

### 4.9 Immunohistochemistry

Tissue specimens were fixed in 10% formalin for 24 h and paraffin embedded. IHC staining was performed on 4‐µm‐thick paraffin sections, which were deparaffinized in xylene and alcohol. Antigen retrieval was performed using citrate buffer (pH 6.0). Sections were blocked in 3% H_2_O_2_ for 10 min and probed with primary antibodies at 4°C overnight in a moist chamber. Slides were rinsed with PBS and incubated with universal immuno‐peroxidase polymer anti‐rabbit or anti‐mouse antibodies (#H2008, #2004; Nichirei Biosciences), and visualized by 3,3′ diaminobenzidine tetrahydrochloride substrate (DAB; #K5007; Dako).

For quantification of staining, tissue specimens were scanned and analyzed using the Vectra software (PerkinElmer Inc., Waltham, USA). For each core, three images of representative areas were analyzed. IHC scoring was performed using Histoscore (*H*‐score) calculated by the image analysis system, which included a semiquantitative assessment of both fraction of positive cells and intensity of staining. The intensity score was defined as no staining (score 0), weak (score 1), moderate (score 2), or strong (score 3) staining. The fraction score was based on the proportion of positively stained cells (0–100%). The intensity and fraction scores were then multiplied to obtain the *H*‐score of 0–3, which were reviewed by two experienced gynecological pathologists. OC tissues were divided into high and low CLK2 expression groups based on the cutoff value determined by ROC curve.

### 4.10 Immunofluorescence

For immunofluorescence assay, cells on confocal dishes were fixed with 4% paraformaldehyde for 20 min at RT and blocked with 1% bovine serum albumin (#ST023; Beyotime) in PBS for 2 h at RT. Cells were then incubated with primary antibodies overnight at 4°C. After incubation, the cells were washed in PBS four times and stained with Alexa Fluor 488‐conjugated anti‐rat IgG (#A21208; Thermo Fisher) and Alexa Fluor 555‐conjugated anti‐rabbit IgG (#A31572; Thermo Fisher) secondary antibodies for 1 h at RT. After washing in PBS four times, the cell nuclei were counterstained with 4′,6‐diamidino‐2‐phenylindole (DAPI, #D1306; Thermo Fisher). Confocal imaging was performed using a confocal laser scanning microscope (LSM 880; Carl Zeiss).

### 4.11 RNA extraction and quantitative real‐time PCR

Total RNA was extracted using TRIzol reagent (#15596026; Thermo Fisher) and quantified using a Nanodrop 2000 spectrophotometer (Thermo Fisher). Total RNA (2 µg) was reversely transcribed using GoScript™ Master Mix (#A2800; Promega). The mRNA levels were detected by Color SYBR Green qPCR Mix (#A0012; EZBioscience) and performed on a LightCycler 480 System (Roche Diagnostics, Basel, Switzerland). GAPDH was used as a control. Primer sequences are listed in Table S5.

### 4.12 Western blotting

Cells were collected and lysed in RIPA lysis buffer (#P0013C; Beyotime). The protein concentrations was measured by a bicinchoninic acid assay (#23227; Thermo Fisher). Approximately 20 µg of total protein extracts were resolved on 8%−15% SDS‐PAGE gels, electrophoresed, and transferred to 0.2 µM polyvinylidenedifluoride membranes (#1620177; Bio‐Rad). After being blocked with 5% nonfat milk in PBS, the membranes were incubated with specific antibodies at 4°C overnight, followed by incubation with a HRP‐conjugated secondary antibody for 1 h at RT. Finally, ECL reagents (#WBKLS0500; Merck Millipore) were used to visualize bands and signals were measured.

### 4.13 Co‐IP assay

For exogenous immunoprecipitation, HEK293T cells were transfected with the indicated plasmids and lysed 48 h after transfection in NP‐40 lysis buffer (#P0013F; Beyotime) supplemented with protease inhibitors (#P1045; Beyotime) on ice for 15 min. After centrifugation at 4°C and 16000 g for 15 min, the supernatants were isolated and incubated with anti‐Flag magnetic beads (#HY‐K0207; MedChemExpress) or anti‐HA magnetic beads (#HY‐K0201; MedChemExpress) at 4°C overnight. The beads were washed with NP‐40 lysis buffer six times before immunoblotting was performed.

For endogenous immunoprecipitation, A2780 cells were harvested and incubated with 2 µg antibodies overnight at 4°C. Protein A/G magnetic beads (#HY‐K0202; MedChemExpress) were then added for 6 h at 4°C. Next, the beads were washed with NP‐40 buffer six times. Finally, the proteins were dissolved in 1×SDS‐PAGE loading buffer, boiled for 10 min at 100°C and analyzed by Western blotting.

For deubiquitination assays, HEK293T cells were transfected with the aforementioned plasmids for 48 h. Before the collection of cell lysates, cells were treated with MG132 (10 µM, #HY‐13259; MedChemExpress) for 6 h. Cell lysates were then subjected to exogenous immunoprecipitation experiments as described above.

A detailed list of antibodies used can be found in Table S6.

### 4.14 Protein half‐life assay

To determine the half‐life of CLK2, A2780 cells were treated either with PBS, cisplatin (6.67 µM) or p38i (10 µM, VX702, #A8687; APExBio) plus cisplatin. After 24 h, cells were treated with 20 µg/mL CHX (#C7698; Sigma–Aldrich) at the indicated time points, and then were collected for Western blot analysis.

### 4.15 Orthotropic xenograft models

For xenograft models, 1 × 10^6^ A2780‐PR Ctrl and A2780‐PR CLK2‐KO cells were subcutaneously inoculated into 5‐week‐old female BALB/c‐nu mice (Guangdong Medical Laboratory Animal Center, Guangzhou, China), respectively. After 2 weeks of inoculation, cisplatin (5 mg/kg) was administered intraperitoneally every 3 days for 2 weeks. Tumor volumes were measured every 3 days. All mice were euthanized after 4 weeks of inoculation, and the xenograft tumors were isolated, photographed, and weighted.

### 4.16 Establishment and passage of PDX models

Under protocols approved by Institutional Review Board and patients’ informed consents, a total of 35 clinical samples were collected and subcutaneously implanted in BALB/c‐nu mice between August 2017 and July 2020, and their host patient characteristics (including age, FIGO stage, family history, histopathological type) were shown (Table S7). Once the tumor tissues were resected from patients with suspected or confirmed ovarian epithelial cancer, they were soaked in sterile DMEM containing 10% FBS and 1% penicillin/streptavidin under 4°C. Within 2 h, samples were washed twice with PBS, cut into 3 × 3 × 3 mm^3^ pieces, and implanted into 5‐week‐old female BALB/c‐nu mice. After postoperative pathological results were obtained, we would mark out and euthanize the mice corresponding to the patients who were not high‐grade serous carcinomas. Mice were sacrificed if there was no evidence of tumor development after a period of 6 months, or reached one of the following conditions: a 40% bodyweight loss, being infective, or moribund. In mice with evidence of tumor growth, weight and tumor volume were evaluated by calipers to assess tumor development. Euthanasia was performed as soon as the tumor xenografts reached a volume of 1500 mm^3^. Following euthanasia, tumors were harvested and cut into 3 × 3 × 3 mm^3^ pieces to be passaged into new mice as serial transplantations. All procedures were conducted in accordance with the Declaration of Helsinki, and all animal experiments conformed to the Welfare of Experimental Animals guidelines at Sun Yat‐sen University.

### 4.17 Drugs treatment

Once the PDX tumors grew to a volume of 100−300 mm^3^, mice were divided into groups randomly and treated with different drugs. Based on the mice's weight, TG003 (30 mg/kg) and Olaparib (50 mg/kg) were administered via intraperitoneal (i.p.) injection every 2 days. Olaparib was administered 30 min prior to TG003. Cisplatin (5 mg/kg) was administered via i.p. injection twice a week. The endpoint for tumor transplantation was determined when a tumor reached 1.5 cm in any dimension. To determine the therapeutic efficacy of the drugs in the PDX models, tumor volumes were monitored using calipers every 2−3 days and calculated using the equation (*L* × *W* ^2^)/2, where *L* stands for length and *W* for width. Four weeks after treatment, mice were euthanized, and the tumors and some organs were harvested for future analysis.

For in vivo treatment, Olaparib was suspended in 4% dimethyl sulfoxide (DMSO; #D806648; Macklin) + 30% PEG300 (#HY‐Y0873; MedChemExpress) in H_2_O. TG003 was suspended in 5% DMSO + 40% PEG300 + 5% Tween80 (#T6336; Macklin) in H_2_O. Cisplatin was dissolved directly in H_2_O. Olaparib and TG003 were stored with parafilm at −20°C and cisplatin was stored at 4°C in dark storage.

### 4.18 Statistics

Data from all experiments were presented as mean ± standard deviation (SD). Student's *t‐*test was utilized to compare the differences between two groups. One‐way analysis of variance was used to compare the differences among three or more groups. The log‐rank test was used to test for differences in survival between the groups. All analyses were performed using GraphPad Prism, version 8 (GraphPad Software Inc., San Diego, CA, USA). *p *< 0.05 was considered statistically significant.

## AUTHOR CONTRIBUTIONS

Y. N. J., S. T. H., L. Z., Y. Z., W. Z., S. J. H., J. D. L., and M. Z. designed the research, wrote the paper, and prepared the figures and tables. X. J. Z., P. P. L., B. Y. P., H. L. X., and M. X. J. designed and performed the experiments. T. W., H. F. G., Y. O., R. Z. L., and W. H. J. provided patient clinical information. All authors read and approved the manuscript.

## CONFLICT OF INTEREST STATEMENT

The authors declare no potential conflicts of interest.

## ETHICS STATEMENT

All samples were obtained with informed consents. Ethical approval was obtained from the Ethics Committee of the Sun Yat‐sen University Cancer Center Institutional Review Board (Approval Number: IRB‐number B2020‐356‐01). The animal experiments of this study were approved by the Institutional Animal Care and Use Committee of Sun Yat‐sen University Cancer Center (Approved Number: L102012017010E). All experiments conformed to all relevant regulatory standards.

## Supporting information

Supporting Information

## Data Availability

All data needed to evaluate the conclusions in the paper are presented in the paper and/or the Supplementary Materials. The authenticity of this article has been validated by uploading the key raw data onto the Research Data Deposit public platform (www.researchdata.org.cn), with the approval RDD number as RDDB2021001624.
