# Supplementary material for: Targeting the Cdc2‐like kinase 2 for overcoming platinum resistance in ovarian cancer
Source: MedComm (2020). 2024 Apr 13;5(4):e537. doi: 10.1002/mco2.537 (PMC11016135; doi:10.1002/mco2.537)
Supplement: Supplementary file 1 — Supporting Information [file MCO2-5-e537-s001.docx]

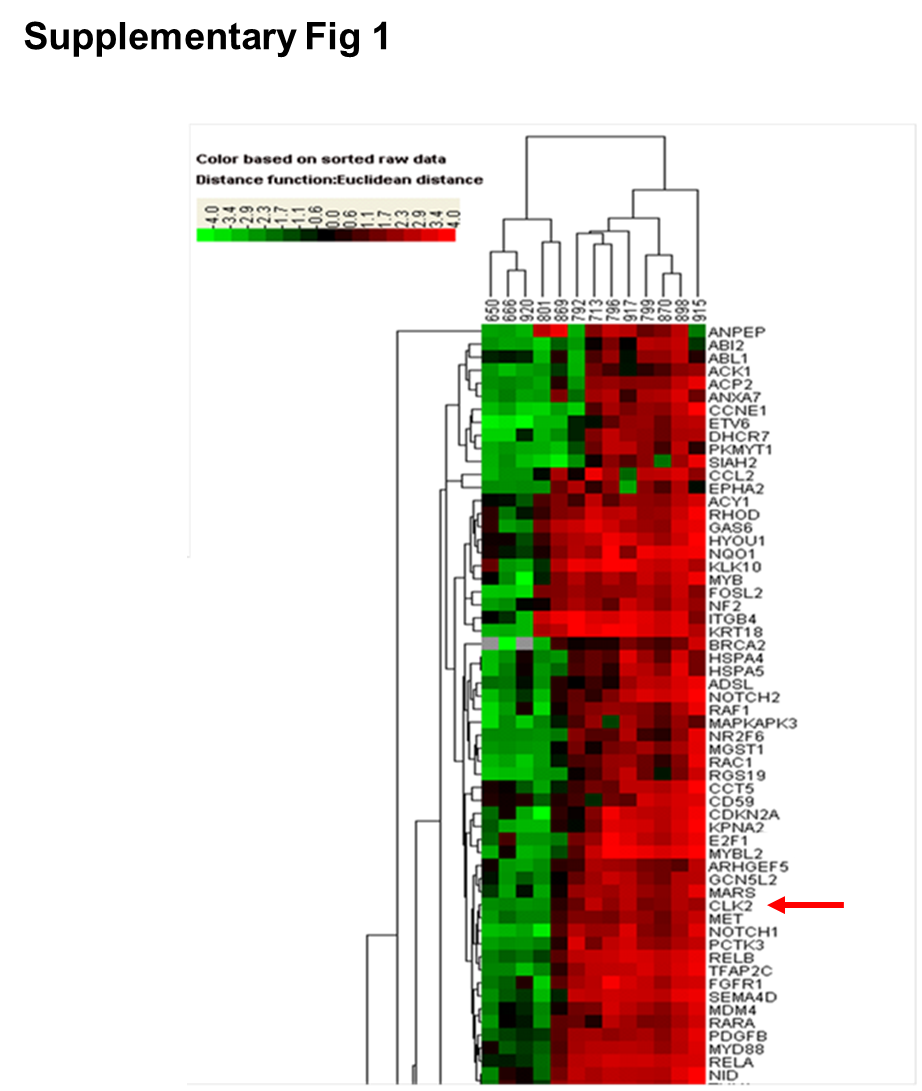


**Supplementary Figure 1 Heatmap of oncogenes differentially expressed in epithelial ovarian cancer (EOC) and human ovarian surface epithelial (HOSE) tissues.**

Three HOSE specimens, one borderline ovarian tumor specimen, and nine EOC specimens were analyzed. Cdc2-like kinase 2 (CLK2) expression was greater in the EOC specimens than that in the HOSE specimens. 650, 666, 920, normal human ovarian surface epithelial tissues; 801, 869, 792, 713, 796, 799, 870, 898, 915, ovarian tumor; 917, borderline ovarian tumor.


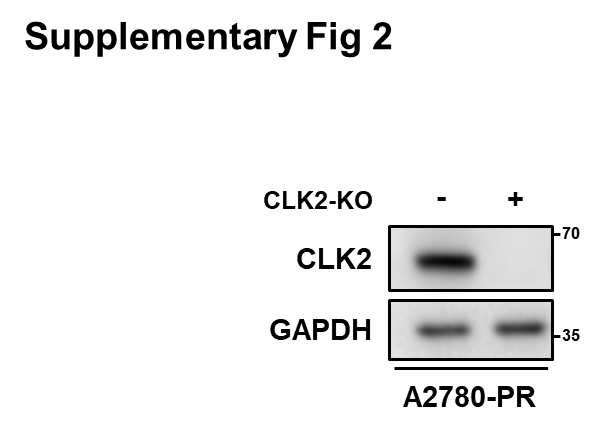


**Supplementary Figure 2 CLK2 knockout efficiency in A2780-PR cells is analyzed by Western blot.**

Platinum-resistant cell lines A2780 (A2780-PR) were transfected with CLK2 single-guide RNA (sgRNA) plasmid. 48 hours after transfection, cells were selected by G418 and were sorted into 96-well plates. Single-cell clones were expanded and validated as CLK2 knockout clone by Western blot.


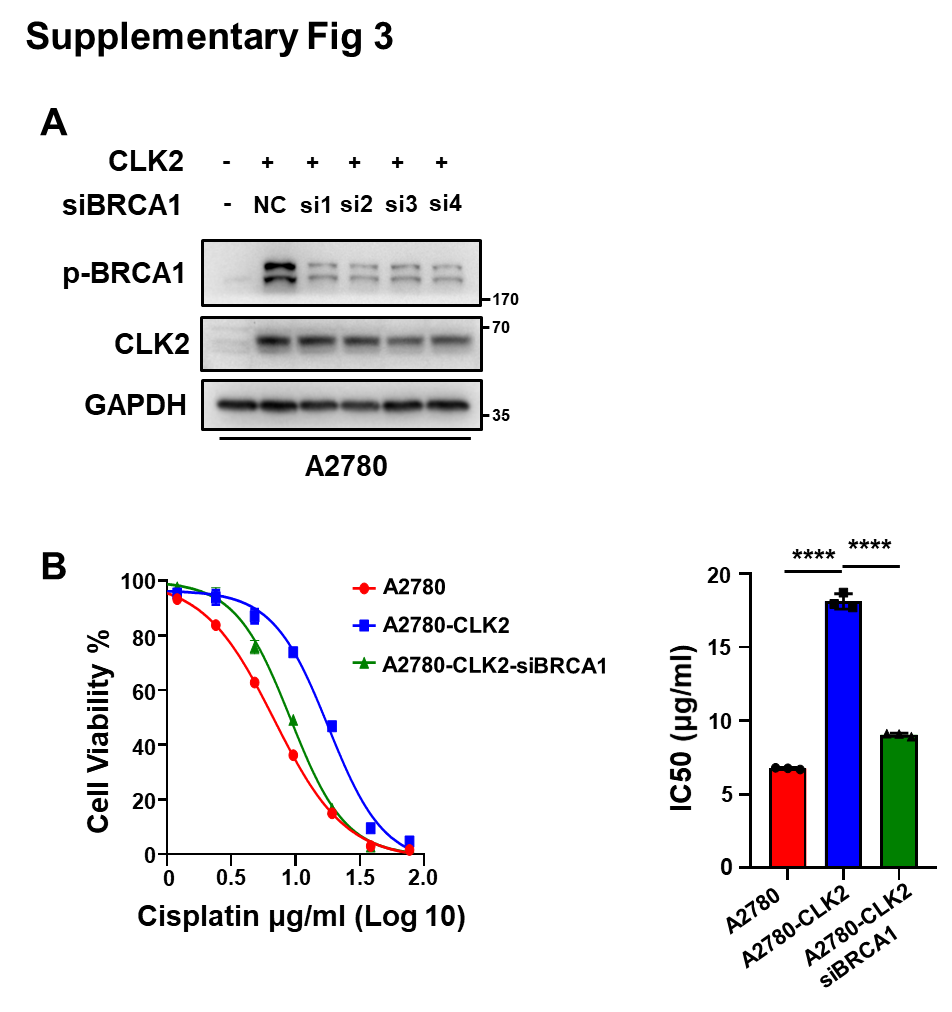


**Supplementary Figure 3 Silencing BRCA1 reverses the enhancement of CLK2-promoted platinum resistance.**

**A** Western blot analyses of protein expressions in A2780 or A2780-CLK2 cells transfected with NC or siBRCA1.

**B** IC_50_ values for cisplatin in CCK8 assays in A2780, A2780-CLK2, A2780-CLK2-siBRCA1 cells (left panel). Cells were treated with cisplatin under a range of concentrations as indicated. IC_50_ values were calculated by determining the concentration needed to inhibit half of the maximum biological response of cisplatin. Quantitation of IC_50_ values was based on the CCK8 assays result (right panel). *****p* < 0.0001. *p* values were determined by Student’s *t* test.


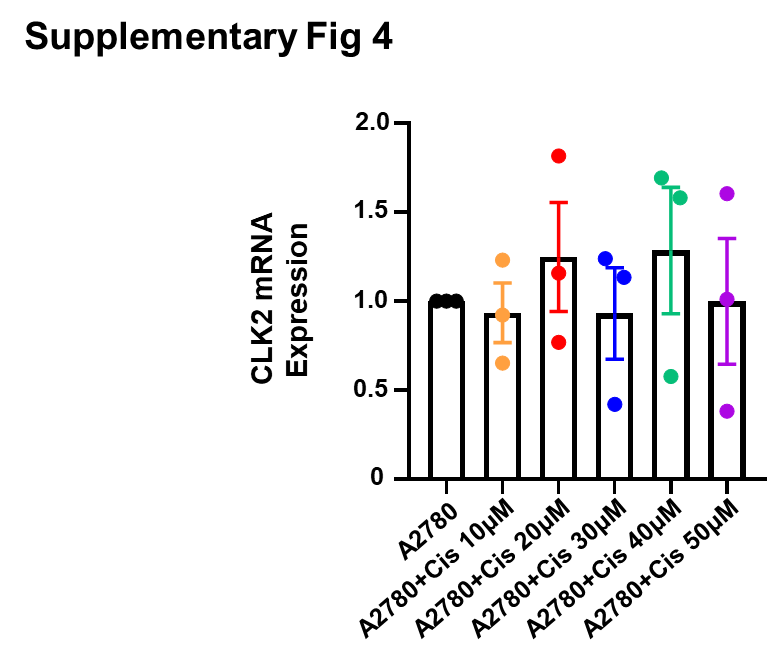


**Supplementary Figure 4 The mRNA expression of CLK2 was not regulated upon cisplatin treatment.**

CLK2 mRNA levels in A2780 cells treated with cisplatin at multiple concentrations were measured by qRT-PCR. Data was shown as mean ± SD.


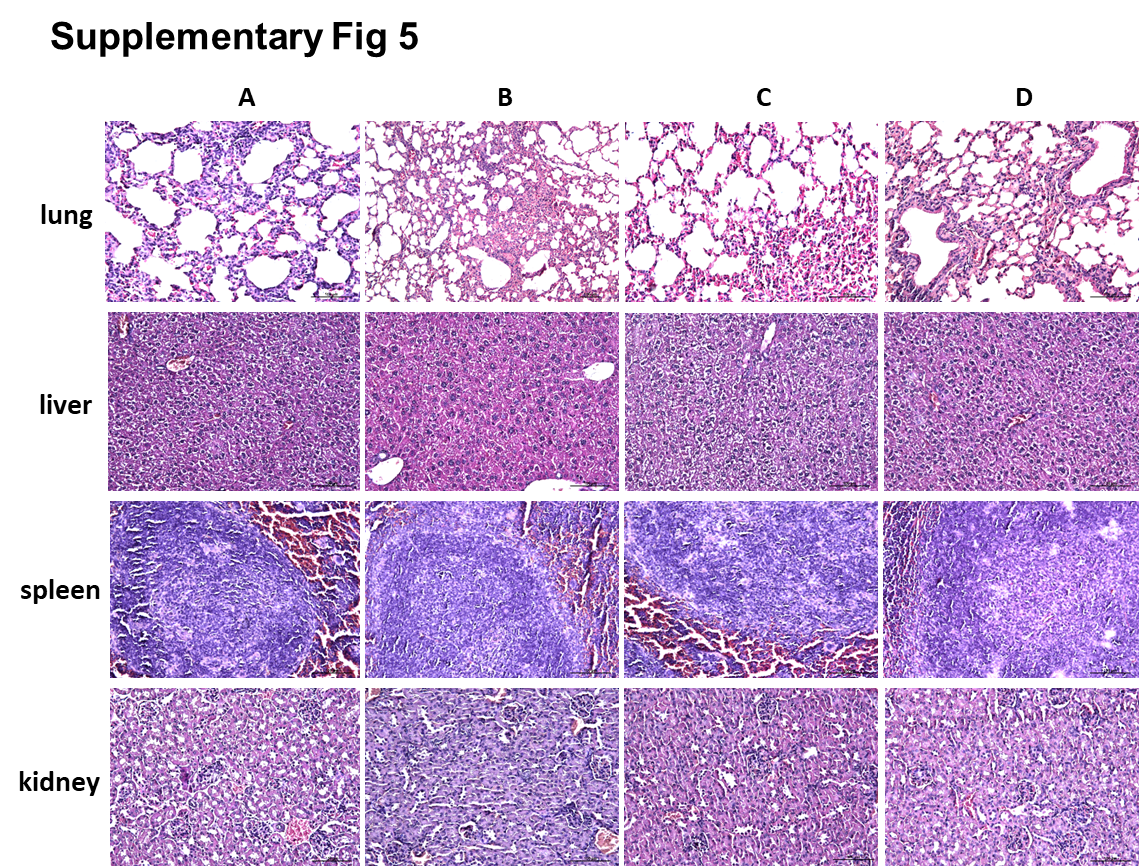


**Supplementary Figure 5 No tissue damage are observed in different treatment groups.**

Hematoxylin and eosin (H&E) staining of lung, liver, spleen and kidney sections from mice carrying PDX. Scale bar for the images at ×20, 100 μm.


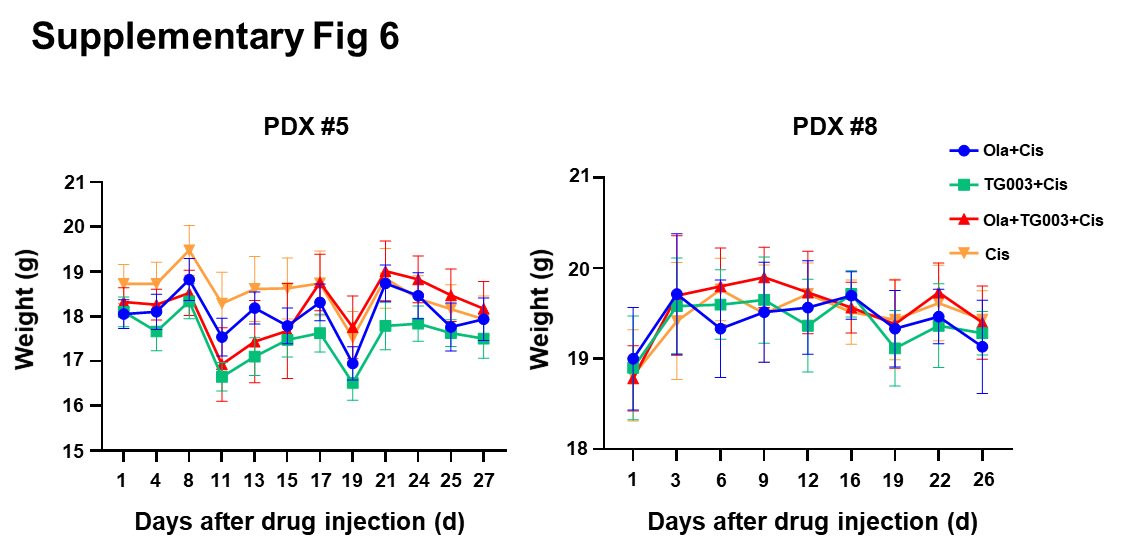


**Supplementary Figure 6 Different treatments have little effect on the mice’s weight.**

Bodyweights of nude mice bearing #5 (left panel) and #8 (right panel) patient-derived-xenografts (PDX) tumors were presented as mean ± SD.

**Supplementary** **Table 1** Clinicopathological characteristics in patients with OC.

| **Characteristics** | **Case (%)** |
| --- | --- |
| Age (years) ^†^ | 52.45±9.940 |
| < 60 | 72 (74.2) |
| ≥ 60 | 25 (25.8) |
| FIGO stage |  |
| IA | 2 (2.06) |
| IB | 1 (1.03) |
| IC | 7 (7.22) |
| IIA | 3 (3.09) |
| IIB | 8 (8.25) |
| IIIA | 0 (0.0) |
| IIIB | 1 (1.03) |
| IIIC | 62 (63.92) |
| IV | 13 (13.40) |
| PFI (months) ^‡^ |  |
| ≤ 6 | 25 (25.77) |
| > 6 | 72 (74.23) |
| CLK2 expression ^§^ |  |
| Low | 60 (61.86) |
| High | 37 (38.14) |
| Recurrence status |  |
| No | 45 (46.39) |
| Yes | 52 (53.61) |
| Survival status |  |
| Alive | 58 (59.79) |
| Death | 39 (40.21) |
| Progression-free survival (months) ^†^ | 29.37±29.95 |
| Overall survival (months) ^†^ | 42.81±28.78 |

^†^ These values were presented as mean with standard deviation (SD).

^‡^ PFI, platinum-free interval.

^§^ The CLK2 expressions were examined in 97 ovarian cancer (OC) patients by immunohistochemistry and the cutoff value was determined using receiver operating characteristic (ROC) curve.

**Supplementary** **Table 2** Correlations between CLK2 expression and clinicopathological features of patients with OC.

| **Clinical variables** | **CLK2 expression ^†^** | | ***p* value ^‡^** |
| --- | --- | --- | --- |
|  | **Low (%) n = 60** | **High (%) n = 37** |  |
| Age (years) |  |  | 0.463 |
| < 60 | 43 (71.7) | 29 (78.4) |  |
| ≥ 60 | 17 (28.3) | 8 (21.6) |  |
| FIGO stage |  |  | 0.313 |
| I-II | 11 (18.3) | 10 (27.0) |  |
| III-IV | 49 (81.7) | 27 (73.0) |  |
| PFI (months) ^§^ |  |  | **0.033** |
| ≤ 6 | 11 (18.3) | 14 (37.8) |  |
| > 6 | 49 (81.7) | 23 (62.2) |  |
| Recurrence |  |  | **0.030** |
| No | 33 (55.0) | 12 (32.4) |  |
| Yes | 27 (45.0) | 25 (67.6) |  |

^†^ The CLK2 expressions were examined in 97 OC patients by immunohistochemistry and the cutoff value was determined using ROC curve.

^‡^ The *p* values were calculated by chi-squared (χ^2^) test. The *p* values in bold indicated significance (*p* < 0.05).

^§^ PFI, platinum-free interval.

**Supplementary Table 3** Univariate and multivariate analyses of prognostic parameters in patients with OC.

| **Clinical variables** | **Subset** | **HR (95% CI) ^†^** | ***p* value ^‡^** |
| --- | --- | --- | --- |
| **Overall survival** |  |  |  |
| Univariate analysis |  |  |  |
| Age (years) | < 60 vs ≥ 60 | 0.776 (0.398-1.512) | 0.456 |
| FIGO stage | I-II vs III-IV | 4.610 (1.417-15.000) | **0.011** |
| CLK2 expression ^§^ | Low vs High | 0.776 (0.383-1.572) | 0.482 |
| Multivariate analysis |  |  |  |
| FIGO stage | I-II vs III-IV | 4.610 (1.417-15.000) | **0.011** |
| **Progression-free survival** |  |  |  |
| Univariate analysis |  |  |  |
| Age (years) | < 60 vs ≥ 60 | 0.804 (0.445-1.454) | 0.471 |
| FIGO stage | I-II vs III-IV | 2.539 (1.140-5.655) | **0.023** |
| CLK2 expression | Low vs High | 1.895 (1.085-3.311) | **0.025** |
| Multivariate analysis |  |  |  |
| FIGO stage | I-II vs III-IV | 2.539 (1.140-5.655) | **0.023** |
| CLK2 expression | Low vs High | 2.941 (1.309-6.611) | **0.009** |

^†^ HR (Hazard Ratio) and *p* values were calculated using univariate or multivariate Cox proportional hazards regression. 95% CI, 95% confidence interval. HR > 1 indicated that the clinical variable is a risk factor.

^‡^ The *p* values in bold indicated significance (*p* < 0.05).

^§^ The CLK2 expressions were examined in 97 OC patients by immunohistochemistry and the cutoff value was determined using ROC curve.

**Supplementary Table 4** Sequences of siRNAs

| **siRNA** | **Sequences** |
| --- | --- |
| siBRCA1-1 | CAGCUACCCUUCCAUCAUA |
| siBRCA1-2 | UAUAAGACCUCUGGCAUGAAU |
| siBRCA1-3 | CAGCAGUUUAUUACUCACU |
| siBRCA1-4 | ACCAUACAGCUUCAUAAAU |
| negative control | UUCUCCGAACGUGUCACGUTT |

**Supplementary Table 5** Primers for real-time PCR

| **Genes** | **Forward** | **Reverse** |
| --- | --- | --- |
| *CLK2* | AAGCAGTTATGATGATCGTTCGT | GGCTATAATCGTTGCGTCTGTAG |
| *GAPDH* | GAGTCAACGGATTTGGTCGT | GACAAGCTTCCCGTTCTCAG |

**Supplementary Table 6** Antibodies for IHC, WB, IP and IF

| **Target protein** | **IHC** | **WB** | **IP** | **IF** |
| --- | --- | --- | --- | --- |
| CLK2 | 1:200, Sigma #HPA055366 | 1:1000, Abcam #ab188141 | #sc-393909 | 1:50, #sc-393909 |
| BRCA1 | 1:400, Abcam #ab213929 | 1:1000, Abcam #ab16780 | Abcam #ab245330 |  |
| p-BRCA1 | 1:200, Abcam #ab47325 | 1:1000, Sigma #SAB4300192 |  | 1:50, Abcam #ab90528 |
| p38 |  | 1:1000, Abcam #ab170099 | Abcam #ab170099 |  |
| RNF8 |  | 1:500, Proteintech #14112-1 | Proteintech #14112-1 |  |
| DNA Damage Antibody Sampler Kit |  | CST #9947 |  |  |
| HA |  | 1:1000, Proteintech #51064-2 |  |  |
| Flag |  | 1:1000, CST #14793 |  |  |
| Myc |  | 1:2000, Proteintech #60003-2 |  |  |
| GAPDH |  | 1:5000, CST #5174 |  |  |

**Supplementary Table 7** Clinicopathological features of OC patients whose tumors were transplanted and established as patient-derived-xenografts in mice.

| **Case** | **Age (y)** | **Family history** | **Histopathological type** | **FIGO Stage** |  |
| --- | --- | --- | --- | --- | --- |
| #017 | 43 | 0 | high-grade serous ovarian adenocarcinoma | IIB |  |
| #024 | 49 | 0 | high-grade serous ovarian adenocarcinoma | IIB |  |
| #026 | 59 | 1 | high-grade serous ovarian adenocarcinoma | IVB |  |
| #L01 | 45 | 0 | high-grade serous ovarian adenocarcinoma | IIIC |  |
| #001 | 60 | 1 | clear cell carcinoma | IA |  |
| #004 | 48 | 0 | endometrioid adenocarcinoma | IIB |  |
| **#5** | 46 | 0 | high-grade serous ovarian adenocarcinoma | IIIC |  |
| #006 | 49 | 0 | high-grade serous ovarian adenocarcinoma | IIIC |  |
| #009 | 60 | 0 | high-grade serous ovarian adenocarcinoma | IIIA |  |
| #028 | 40 | 1 | clear cell carcinoma | IA |  |
| #051 | 51 | 0 | clear cell carcinoma | IIIC |  |
| #054 | 56 | 1 | high-grade serous ovarian adenocarcinoma | IIIC |  |
| **#8** | 53 | 1 | high-grade serous ovarian adenocarcinoma | IIIC |  |
| #3 | 66 | 0 | high-grade serous ovarian adenocarcinoma | IIIC |  |
| #058 | 30 | 0 | ovarian sex cord stromal tumor | IA |  |
| #061 | 48 | 0 | endometrioid adenocarcinoma | IIA |  |
| #069 | 61 | 0 | clear cell carcinoma | IIB |  |
| #F01 | 44 | 1 | hepatocellular carcinoma ovarian metastasis | IVB |  |
| #F02 | 53 | 0 | clear cell carcinoma | IC |  |
| #F03 | 62 | 0 | high-grade serous ovarian adenocarcinoma | IVB |  |
| #F04 | 45 | 0 | high-grade serous ovarian adenocarcinoma | IB |  |
| #F05 | 23 | 0 | serous borderline epithelial tumors | IIB |  |
| #F06 | 50 | 0 | gastrointestinal adenocarcinoma |  |  |
| #F07 | 45 | 1 | endometrioid adenocarcinoma | IC |  |
| #F08 | 68 | 1 | high-grade serous ovarian adenocarcinoma | IIIC |  |
| #F09 | 26 | 0 | clear cell carcinoma | IC |  |
| #F10 | 32 | 0 | serous borderline epithelial tumors | IIIC |  |
| #F11 | 58 | 0 | clear cell carcinoma | IIIC |  |
| #F12 | 66 | 1 | mucinous adenocarcinoma | IIIC |  |
| #F13 | 53 | 1 | high-grade serous ovarian adenocarcinoma | IIIC |  |
| #F14 | 48 | 1 | high-grade serous ovarian adenocarcinoma | IIIC |  |
| #F15 | 55 | 1 | high-grade serous ovarian adenocarcinoma | IIIA |  |
| #F16 | 60 | 1 | endometrioid adenocarcinoma | IIIA |  |
| #L1 | 56 | 0 | serous borderline epithelial tumors | IIIC |  |
| #R2 | 72 | 0 | high-grade serous ovarian adenocarcinoma | IIIC |  |
